# Extremes on the benign-malignant tumour spectrum: Distinct transcriptomic landscapes between two common canine perianal neoplasms based on the hallmarks of cancer

**DOI:** 10.1371/journal.pone.0351773

**Published:** 2026-06-22

**Authors:** Alexander F. H. Haake, Alina K. Loriani Fard, Vladimir M. Jovanovic, Michael Hummel, Sandro Andreotti, Achim D. Gruber

**Affiliations:** 1 Department of Veterinary Medicine, Institute of Veterinary Pathology, Freie Universität Berlin, Berlin, Germany; 2 Department of Mathematics and Computer Science, Bioinformatics Solution Center, Freie Universität Berlin, Berlin, Germany; 3 Institute of Pathology, Charité – Universitätsmedizin Berlin, Berlin, Germany; Sichuan University, CHINA

## Abstract

The hallmarks of cancer describe stepwise changes in cellular functions and molecular pathways of tumour cells that undergo malignant progression. Although 14 hallmarks have been proposed, their manifestation at the molecular level across different tumour types remains incompletely understood. Here, we hypothesised that hallmark-associated transcriptomic signatures reflect the contrasting biological behaviours of a pair of a benign and a malignant tumour. To this end, we compared the two most common canine perianal tumours, the highly malignant apocrine gland anal sac adenocarcinoma (AGASAC) and the benign hepatoid gland adenoma (HGA). Spontaneous primary tumours were analysed using RNA-Seq (AGASAC n = 11, HGA n = 10) and a direct mRNA hybridisation panel (AGASAC n = 28, HGA n = 12). Large transcriptomic differences were observed in both the number of differentially expressed genes and enriched pathways. Surprisingly, transcriptomic differences based on the hallmarks of cancer were less pronounced that anticipated, particularly for “activating invasion and metastasis” and “inducing or accessing vasculature”. Only the HGAs showed a significant enrichment for “deregulating cellular energetics”. However, our data point towards a contrasting immune landscape between the compared tumours and a subgrouping of AGASACs associated with the inflammatory landscape. Overall, the transcriptomes of these two benign-malignant tumour extremes on the tumour spectrum contrasted each other in select aspects of the hallmarks. Still, their malignant or benign behaviours could not generally be mirrored on the mRNA level.

## Introduction

Molecular characterisation of canine tumours can open up new insights into comparative understanding of tumour biology, tumour-specific properties, as well as novel strategies for improved treatment in this species. In both humans and animals, pan-cancer research has cumulated in a multifaceted perspective on biological properties leading to malignant transformation. Specifically, fourteen comprehensible concepts, proposed by Hanahan and Weinberg as the hallmarks of cancer, describe acquired features that promote and sustain tumourigenesis [1–3]. It is postulated that nearly all tumours exhibit a multistep acquisition of at least six of the eight “hallmark capabilities”. The succession of obtaining these varies both in time point and their molecular mechanism. As genetic alterations can affect multiple molecular processes, a single genetic aberration can convey various capabilities concurrently. Furthermore, six “enabling characteristics” or “emerging hallmarks” likely aid to the development and maintenance of cancer. Purposely, the concept of these hallmarks should be applicable to all tumours, independent of their cellular origin or species.

Among the extremes on the benign-malignant tumour spectrum are the two most common perianal tumours in dogs, the hepatoid gland adenoma (HGA) and apocrine gland anal sac adenocarcinoma (AGASAC). Despite their similar location and initial clinical impression, they exhibit contrasting biological and clinical behaviours. Specifically, the HGA almost always will demonstrate benign behaviour and in most cases, simple surgical removal is curative [4]. In stark contrast, the highly malignant AGASAC often develops early invasion and metastasis to the locoregional lymph nodes even in small tumours not yet detected [4–10]. Metastatic rates at time of clinical presentation of up to 79% are reported [11]. Distant metastases may also occur [5,11–13]. Standard therapy consists of surgical extirpation of the affected anal sacs and lymph nodes. Despite additional adjunct therapies including chemo- and radiotherapy, survival rates generally remain poor [5,11,14–22]. Thus, these perianal tumours represent an intriguing pair for comparison, as they exhibit distinct and consistent biological behaviours, permitting an inter-entity contrast that serves as a proof of principle for identifying hallmark features of cancer. This approach differs from previous intra-entity comparisons, where malignant tumours have been compared to benign tumours of the same cellular origin [23–27].

We hypothesised that AGASACs would particularly feature transcriptomic profiles linked to early aggressive metastatic behaviour, while HGAs would display profiles commonly linked to benign growth, including general tumour development, yet lack those related to malignancy. Furthermore, we speculated that two distinct transcriptomic landscapes based on the hallmarks of cancer would be evident, each characteristic of the distinct biological behaviour of the two tumour entities. As part of improved tumour understanding, the transcriptomic differences between AGASAC primary tumours and their corresponding nodal metastases were also investigated. Finally, we asked whether the data could potentially further novel therapeutic strategies for the commonly lethal AGASAC [28].

To this end, we analysed archival formalin-fixed, paraffin-embedded (FFPE) tumours from spontaneously affected pet dogs with two distinct technologies to establish whether specific transcriptomic differences mirror, predict, or explain the extremely different biological behaviours of HGAs and AGASACs. Specifically, RNA sequencing (RNA-Seq) allowed for a whole transcriptome landscaping approach, while a direct mRNA hybridisation panel, nCounter^®^, enabled a more focussed view on immuno-oncological processes.

## Materials and methods

### Tissue samples

FFPE tissue samples of AGASACs (n = 33; 28 primary tumours, 5 nodal metastases) and HGAs (n = 12) from pet dogs were retrospectively selected from routinely archived cases between 2006 and 2021 at the Institute of Veterinary Pathology, Freie Universität Berlin. Tumours were diagnosed histopathologically by a board-certified veterinary pathologist on 2 μm-thick sections routinely stained with haematoxylin and eosin.

The dogs were privately owned and were not associated to animal experiments. Tumours had been surgically excised for curative and diagnostic reasons only. Owners had given their consent to the use of tissues for research purposes. The work was ethically approved (decision StN 010/23 of the State Office for Health and Social Affairs, Berlin).

Selection criteria included a perianal location, specifically the left or right anal sac for AGASACs, and also included the tail root for HGAs. The dogs were at least 8 years old (span: 8–15, mean: 10.76). The selected tumours were solid and samples with ulceration and suppurative inflammation, larger areas of necrosis, fibrosis, or desmoplasia were excluded. See S1 Table for further data on the employed samples. In total, the archived samples reviewed prior to selection included 188 AGASAC and 242 HGA submissions.

The nodal metastases stemmed from five cases diagnosed with AGASAC (a1 to a5), where sublumbar lymph node metastases from the same dog had been submitted simultaneously.

### RNA extraction and quality control

After trimming away tumour-adjacent tissue with a scalpel, five 10 μm FFPE scrolls were prepared from entire cross sections, collected in sterile centrifuge tubes, and stored at −80 °C. RNase AWAY^®^ (Thermo Fisher Scientific Inc., USA) was applied to all surfaces, gloves, and instruments before RNA isolation.

Total RNA was extracted in randomised extraction batches (S2 Table) with the PureLink^TM^ FFPE Total RNA Isolation Kit (Thermo Fisher) according to the manufacturer’s guidelines. Total RNA concentrations were measured with a UV-VIS spectrophotometer NanoDrop^TM^ 2000c (Thermo Fisher) and the quality of the total RNA was determined with an Agilent 5200 Fragment Analyzer (Agilent Technologies, Inc., USA) employing the DNF-471F33 - SS Total RNA 15nt - FFPE Illumina DV200 method mode. For data on RNA quality see S2 Table. Only samples with a DV_200_ (percentage of RNA fragments over 200 nucleotides [nt] in length) [29] of > 40% were chosen. Total RNA was treated with DNase I (Thermo Fisher).

### RNA sequencing (RNA-Seq) and bioinformatic analysis

The RNA from 25 samples (21 cases), i.e., 10 HGAs (10 cases) and 15 AGASACs (11 primary tumours and 4 nodal metastases; 11 cases), was sequenced with QuantSeq 3' (Lexogen GmbH, Vienna, Austria) at Lexogen Services. Additionally, three technical replicates from each three HGA samples (h2, h24, h28) were obtained and sequenced. DNase I-treated total RNA (500–1000 ng) was processed with the QuantSeq 3' mRNA-Seq FWD Library Preparation Kit (Lexogen) according to the manufacture’s guidelines (user guide 015UG009V0251) using the low-quality RNA protocol. The quality of the libraries was determined with the Agilent 5300 Fragment Analyzer (DNF-474–33 – HS NGS Fragment 1–6000 bp method mode). The samples were pooled in equimolar ratio and the library pool was quantified using a Qubit^TM^ dsDNA HS assay kit (Thermo Fisher). Sequencing was performed on an Illumina^®^ NextSeq^TM^ 500 system with a SR75 High Output Kit. In total, 0.011 sequencing lanes were used per sample, resulting in an output of 4,025,165–7,548,231 trimmed reads with average read lengths between 63.41 to 71.6 nt.

The FASTQ sequencing files were first pre-processed (adapter trimmed, filtered) using Cutadapt (v1.18) [30] and aligned to the current NCBI Reference Sequence (RefSeq) assembly for the dog (*Canis lupus familiaris*) UU_Cfam_GSD_1.0 (GCF_011100685.1) [31] with STAR aligner (v2.7.11b) [32]. Gene-level counts were generated with two rounds of featureCounts (v2.0.1) [33]. As the mapping of QuantSeq 3' reads is restricted to the 3' end of expressed sequences, insufficient annotations of the 3' UTR may result in the omission of the identification of genuinely expressed transcripts. To mitigate this effect, we employed a second round of featureCounts for previously unassigned mappings against transcripts with a 2 kilobase (kb) extension of their 3' UTR. This 2 kb extension was selected based on our preliminary analyses of read mapping locations downstream of annotated 3' ends on canine tissue samples not used in this experiment, which had also been analysed with QuantSeq 3'. This approach increased the average number of assigned read mappings by 11% (minimum: 9%, maximum: 15%).

Gene counts for technical replicates were aggregated before raw gene counts were normalised and principal component analysis (PCA) ensued with the plotPCA function from the DESeq2 package (v1.28.0) [34]. DESeq2 was then used for differential gene expression (DGE) analysis for the comparison AGASAC primary tumours versus HGAs. Additionally, the AGASAC primary tumours were compared to their corresponding nodal metastases (each n = 4). A batch effect was not evident after visual inspection of the PCA plot with according identification by RNA extraction batches.

The significance thresholds were drawn at a log_2_(fold change) (log_2_FC) of ≤ −1 and ≥ 1 after shrinkage with apeglm [35] and an adjusted p-value (p_adj_) of < 0.05. In calculating the p_adj_, a false discovery rate (FDR) of 0.05 using the Benjamini-Hochberg (BH) procedure [36] was used. The key parameters for the software are listed in S3 Table.

The statistical power of the experimental design, estimated using the RNASeqPower method [37] as implemented in the online application (https://rodrigo-arcoverde.shinyapps.io/rnaseq_power_calc/), was > 0.99. The following parameters were used: feature counts (referred to as sequencing depth in the tool) = 81, coefficient of variation = 0.4, effect = 2, alpha = 0.05, sample size = 25.

### Immuno-oncological nCounter^®^ direct mRNA hybridisation panel

The RNA (150–250 ng) from 45 samples (25 of which had already been analysed with RNA-Seq), i.e., 12 HGAs (10 of which had already been analysed with RNA-Seq) and 33 AGASACs (15 of which had already been analysed with RNA-Seq) – 28 primary tumours and 5 nodal metastases – was hybridised in random order (S1 Table) to the nCounter^®^ Canine IO Panel XT CodeSets (NanoString Technologies, Inc., USA). The extracted RNA stemmed from the same extractions used for RNA-Seq. A 30 probe Panel Plus (S4 Table) was added to the hybridisation following the manufacturer’s hybridisation protocols (manual IDs: MAN-10023–11, MAN-10056–06). This Panel Plus included genes of further interest for these entities stemming from the RNA-Seq analysis. The probes were designed on the basis of the NCBI RefSeq assembly CanFam3.1 (GCF_000002285.3) [38]. Hybridised samples were loaded onto the nCounter^®^ MAX Analysis (NanoString) Prep Station for purification and immobilisation on sample cartridges, transferred to the Digital Analyzer for data collection, and analysed following the manufacturer’s protocol (manual ID: MAN-C0035-08).

Following the workflow described in the manufacturer’s guidelines (manual IDs: MAN-C0019-08, MAN-C0011-04), the reporter library file (RLF) and reporter code count (RCC) files were imported into the nSolver^TM^ 4.0 Analysis Software (NanoString). Quality control and normalisation followed default settings.

### Analysis of nCounter^®^ data with nSolver^TM^

Further analyses were implemented with the R 3.3.2-based Advanced Analysis 2.0 plug-in (v2.0.134) following the manufacturer’s instructions (manual ID: MAN-10030–03) with the recommended statistical settings.

For DGE analysis, this included using a FDR of 0.05 with the Benjamini-Yekutieli (BY) [39] procedure. In a gene panel context, the BY method is preferred, as expression profiles among the selected genes are typically correlated due to their biological interrelatedness. The BY method controls the FDR under arbitrary test dependence, making it well suited for transcriptomic panels where interdependencies exist. Additionally, NanoString recommends the use of BY with nCounter^®^ panels. Main inferences remained stable across an alternative FDR correction method (BH). Differential expression was calculated for AGASAC primary tumours (n = 28) versus baseline of HGA (n = 12). Furthermore, the AGASAC primary tumours were also compared to their corresponding nodal metastases (each n = 5). A clear batch effect was not evident after visual inspection of the PCA plot and heatmap with colouring based on cartridge ID, but the cartridge ID was used as a confounder in the DGE analysis, as recommended, and the sample group was selected as predictor.

For gene set analysis (GSA), a custom annotation file based on the hallmarks of cancer was provided by NanoString’s gene curation team (S5 Table). Gene annotations were adapted from annotations based on the Gene Ontology (GO) [40,41], Kyoto Encyclopaedia of Genes and Genomes (KEGG) [42], and Reactome [43,44] databases and assigned to matching hallmarks (S6 Table). A custom annotation route using an annotation engine was used by NanoString’s gene curation team to assign annotations to the genes included on the Panel Plus based on this research context when possible. Global significance scores (GSS), based on the t-statistics of the differentially expressed genes within a hallmark, were automatically calculated in the GSA module. The GSS indicates how strongly genes in a hallmark differ overall between the AGASACs versus HGAs. The higher the score, the more transcriptional change is seen. The directed GSS (DGSS) also includes the direction, i.e., a positive score indicates an up-regulation in AGASACs relative to HGAs, while a negative score indicates down-regulation. A significance threshold is not provided.

The pathway scoring module automatically summarizes the data from all genes belonging to a hallmark gene set into a single pathway score. It is essentially a first principal component (PC1) of the normalised expression levels of a hallmark gene set. A higher score indicates that the genes of a hallmark are collectively more highly expressed relative to other samples. The user manual MAN-10030–03 includes more information on GSS and pathway scoring.

We had previously shown that there was a strong correlation of gene expression data between QuantSeq 3' and nCounter^®^ [45].

### Interpretation of log_2_FC directions

Differentially expressed genes (DEGs) were calculated based on the comparison of AGASAC primary tumours versus HGAs in DESeq2 and nSolver^TM^. Thresholds were set at log_2_FC ≤ −1 or ≥ 1. Genes with log_2_FC ≥ 1 were considered to be expressed higher, while genes with log_2_FC ≤ −1 were considered to be expressed lower in the AGASAC samples. Vice versa applied to the HGA samples.

### Over-representation analysis for the hallmarks of cancer

Over-represented cancer hallmarks were identified with cancerhallmarks.com [46]. All significantly DEGs from the AGASAC primary tumour versus HGA comparison were split into two distinct sets based on a higher (log_2_FC ≥ 1) or lower (log_2_FC ≤ −1) expression. These two gene sets using the annotated gene symbols were used as independent inputs. The “core cancer hallmark gene set”, comprising 1,574 genes, the “human” species option, and the output as “enrichment with distribution plot” was selected.

Additionally, gene set enrichment analysis on the basis of the Molecular Signatures Database (MSigDB) hallmark gene set collection [47] was chosen in the same online tool. This collection comprises 50 gene sets. Significance thresholds were drawn at p_adj_ ≤ 0.05.

## Results

### Striking differences between AGASAC and HGA samples in the RNA-Seq data

**Fig 1:**
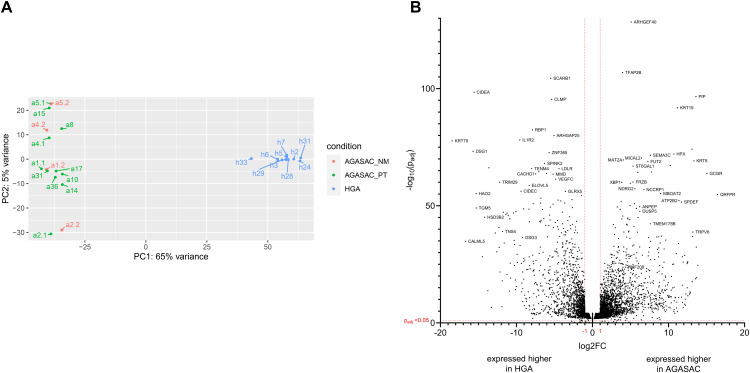
Gene expression data from RNA-Seq. **(A)** Principal component analysis (PCA) plot of the 25 tumour samples analysed with RNA-Seq. PCA was performed on the rlog normalised reads in DESeq2. The HGA samples (“h”) are represented by the turquoise dots, the AGASAC primary tumour samples (“a”) by the green dots. Four primary tumours are labelled as aX.1, lymph node metastases (orange dots) as aX.2. **(B)** Volcano plot of the significantly differentially expressed genes after DESeq2 analysis on the basis of the RNA-Seq data. The red dotted lines represent the significance thresholds (p_adj_ < 0.05; log_2_FC < −1 and > 1). AGASAC, apocrine gland anal sac adenocarcinoma; NM, nodal metastasis; PT, primary tumour; HGA, hepatoid gland adenoma; PC, principal component; FC, fold change; p_adj_, adjusted p-value.

We performed a PCA on rlog normalised RNA-Seq read counts in order to elucidate the differences between the transcriptomes of AGASACs and HGAs in a landscaping approach. The first two principal components (PCs) accounted for 70% of the variance in gene expression (PC1: 65%; PC2: 5%; Fig 1A). All HGA samples were clearly separated from the AGASAC samples. Interestingly, while tight clustering of the HGAs indicated only minor transcriptomic differences among this group, the AGASACs were more dispersed along PC2. Moreover, each of four primary tumours (aX.1) clustered closely with its corresponding lymph node metastasis (aX.2).

DESeq2 analysis identified a total of 3,633 genes as significantly differentially expressed between the AGASAC and HGA groups. Of these, 2,235 genes were higher expressed and 1,398 were lower expressed in the AGASAC group (S7 Table). A volcano plot visualises the pattern of DEGs with select identification of genes with the strongest differences (Fig 1B).

### Genes with strongest differentiation between AGASAC and HGA samples

Among the DEGs, we selected single genes that could best discriminate the tumour entities, potentially also for diagnostic purposes. Ten genes were identified that had high read counts in all samples of one tumour group, but zero in all or most of the other tumour samples (Table 1). Among these, PIP showed high expression in AGASACs (average log_10_ normalised reads = 4.05), and more than 670-fold higher reads level in the AGASAC sample with lowest expression (2,564 reads) compared to the HGA sample with highest expression (3.8 reads). Three of the identified genes (PIP, KRT19, KRT8) had very few reads (< 5 normalised reads in a maximum of four samples) in the HGAs, while another three (CIDEA, DSG3, TRIM29) had very few reads (≤ 2.5 normalised reads in a maximum of one sample) in the AGASACs.

**Table 1:** Ten highly differentiating genes.

Differentiating for	Gene	Normalised reads in HGA samples (mean)	Normalised reads in AGASAC samples (mean)	Difference between means (log_10_-factor)	log_2_FC
AGASAC	PIP	0–3.8 (0.89)	2,564–17,812 (9,860)	4.05	13.61
	KRT19	0–4.6 (0.93)	1,122–3,267 (2,189)	3.37	11.18
	KRT8	0–1.3 (0.13)	668–1192 (880)	3.83	13.33
	GCGR	0	196–4,948 (2,244)	∞	15.05
	QRFPR	0	196–4,974 (2,254)	∞	16.44
HGA	CIDEA	1,155–7,105 (4,769)	0–1 (0.09)	4.72	−15.57
	KRT79	117–7,427 (4,642)	0	∞	−18.44
	DSG1	489–1,256 (804)	0	∞	−15.7
	DSG3	92–321 (160)	0–2.5 (0.22)	2.86	−9.19
	TRIM29	283–959 (483)	0–0.9 (0.08)	3.78	−12.22

Ten genes are displayed that highly differentiate between the apocrine gland anal sac adenocarcinoma (AGASAC) primary tumour and hepatoid gland adenoma (HGA) samples, possibly useful for diagnostic purposes. FC, fold change.

### Only one significantly over-represented hallmark of cancer after using RNA-Seq data

**Fig 2:**
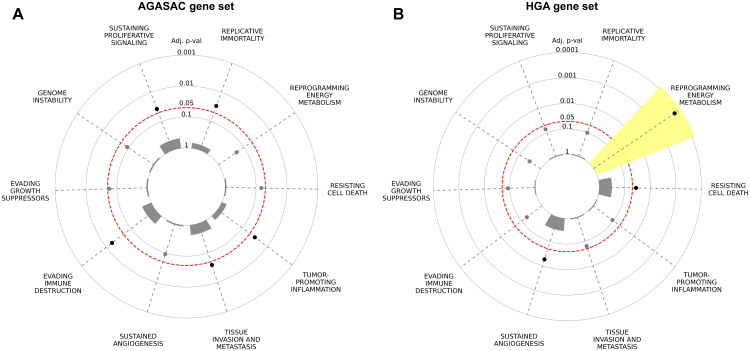
Over-representation analysis for the hallmarks of cancer. Hallmark enrichment plots from cancerhallmarks.com [46] for the **(A)** AGASAC gene set (n = 2,235) and **(B)** HGA gene set (n = 1,398) compared to the core hallmark gene set (n = 1,574 genes). The grey slices indicate no significant enrichment, while the coloured slice shows significant enrichment. The enrichment p-values are displayed as concentric circles, while the red dotted line marks the significance threshold of p_adj_ ≤ 0.05. The dots represent the distribution of genes across the ten hallmarks relative to one another. Black dots denote a greater number of genes (ratio > 1), whereas grey dots indicate hallmarks with comparatively fewer genes (ratio < 1).

Surprisingly, no hallmarks were significantly over-represented in the AGASAC gene set (n = 2,235, Fig 2A) after input into cancerhallmarks.com [46]. However, some hallmarks were more discernible based on the ratios indicating whether a gene set of one hallmark was distributed to a greater extent (ratio > 1) compared to the other hallmarks. This was the case in six hallmarks: “tissue invasion and metastasis” (p_adj_: 0.475, ratio: 1.09), “sustaining proliferative signalling” (p_adj_: 0.475, ratio: 1.1), “evading immune destruction” (p_adj_: 0.475, ratio: 1.36), “tumour-promoting inflammation” (p_adj_: 0.675, ratio: 1.15), and “replicative immortality” (p_adj_: 0.675, ratio: 1.17).

For the HGA gene set (n = 1,398, Fig 2B), only one hallmark was significantly over-represented: “reprogramming energy metabolism” (p_adj_: 3.45e-11, ratio: 2.96). Higher ratios were visible in two hallmarks: “sustaining angiogenesis” (p_adj_: 0.32, ratio: 1.34) and “resisting cell death” (p_adj_: 0.32, ratio: 1.11). See S8 Table for the full output.

**Fig 3:**
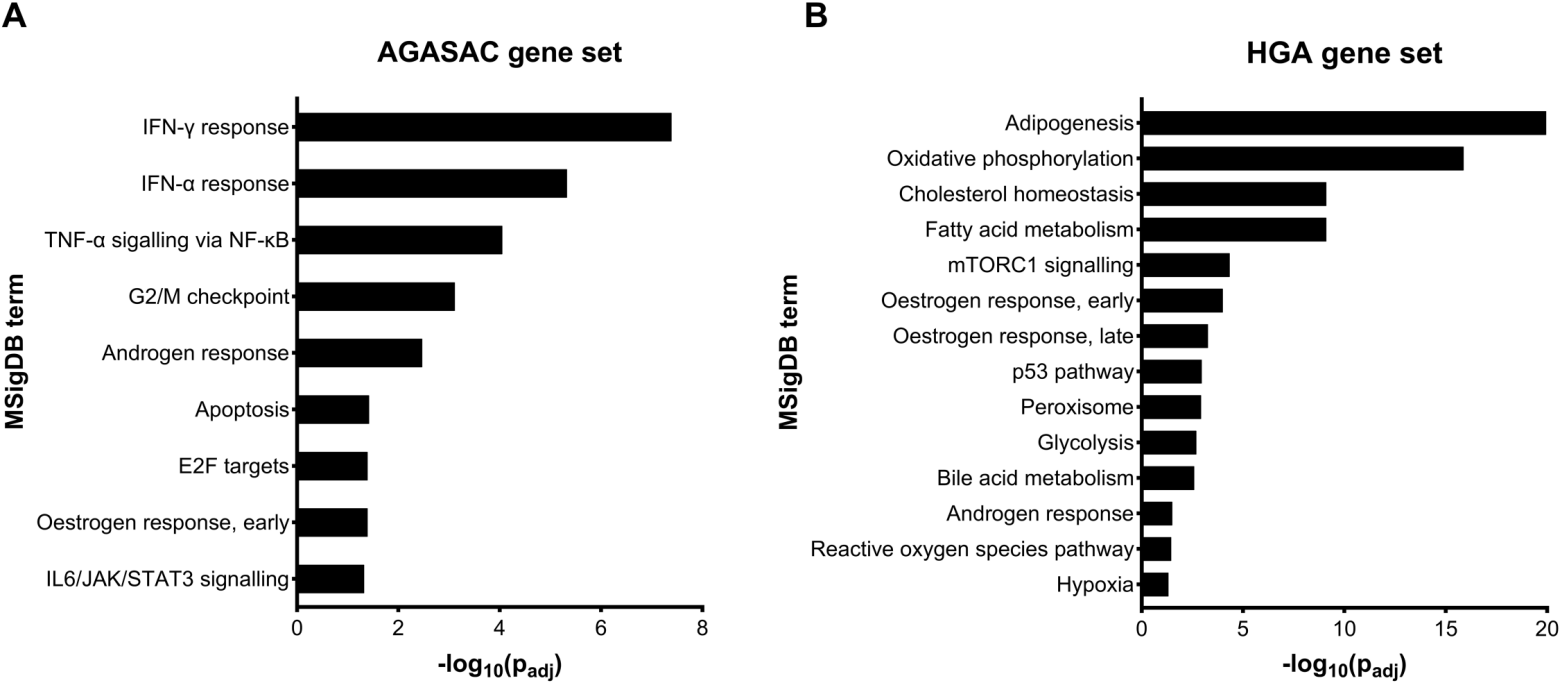
Significantly enriched Molecular Signature Database (MSigDB) terms. Gene set enrichment analysis results from cancerhallmarks.com for the **(A)** AGASAC gene set (n = 2,235) and **(B)** HGA gene set (n = 1,398) compared to the 50 MSigDB hallmark gene sets. AGASAC, apocrine gland anal sac adenocarcinoma; HGA, hepatoid gland adenoma; p_adj_, adjusted p-value.

Using the MSigDB [47,48] hallmark gene sets, we performed gene set enrichment analyses and identified nine significantly enriched terms in the AGASAC gene set (Fig 3A) and fourteen in the HGA gene set (Fig 3B). See S9 Table for the full output.

The nine terms enriched in the AGASAC gene set encompassed “IFN-γ response” (p_adj_: 4.06e-8), “IFN-α response” (p_adj_: 4.73e-6), “TNF-α signalling via NF-κB” (p_adj_: 8.9e-5), “G2/M checkpoint” (p_adj_: 7.66e-4), “androgen response” (p_adj_: 3.39e-3), “apoptosis” (p_adj_: 0.038), “E2F targets” (p_adj_: 0.041), “oestrogen response, early” (p_adj_: 0.041), and “IL6/JAK/STAT signalling” (p_adj_: 0.047).

The fourteen terms enriched in the HGA gene set comprised “adipogenesis” (p_adj_: 1.13e-20), “oxidative phosphorylation” (p_adj_: 1.32e-16), “cholesterol homeostasis” (p_adj_: 7.8e-10), “fatty acid metabolism” (p_adj_: 7.8e-10), “mTORC1 signalling” (p_adj_: 4.55e-5), “oestrogen response, early” (p_adj_: 9.98e-5), “oestrogen response, late” (p_adj_: 5.23e-4), “p53 pathway” (p_adj_: 1.1e-3), “peroxisome” (p_adj_: 1.19e-3), “glycolysis” (p_adj_: 2e-3), “bile acid metabolism” (p_adj_: 2.57e-3), “androgen response” (p_adj_: 0.031), “reactive oxygen species pathway” (p_adj_: 0.035), and “hypoxia” (p_adj_: 0.048).

Both gene sets showed significant enrichment of the “androgen response” and “oestrogen response, early”. “Androgen response” was enriched stronger in the AGASAC gene set, while “oestrogen response, early” was more prominently enriched in the HGA gene set.

### Analysis of the hallmarks of cancer using nCounter^®^ identifies differences in immune landscapes between the AGASAC and HGA

We increased the number of AGASAC primary tumour samples from 15 to 28 to identify possible transcriptomic reasons for the higher variation of samples that was implied in the PCA plot on the RNA-Seq data. One of these AGASAC primary tumours (a3.1) also had a corresponding lymph node metastasis sample (a3.2).

**Fig 4:**
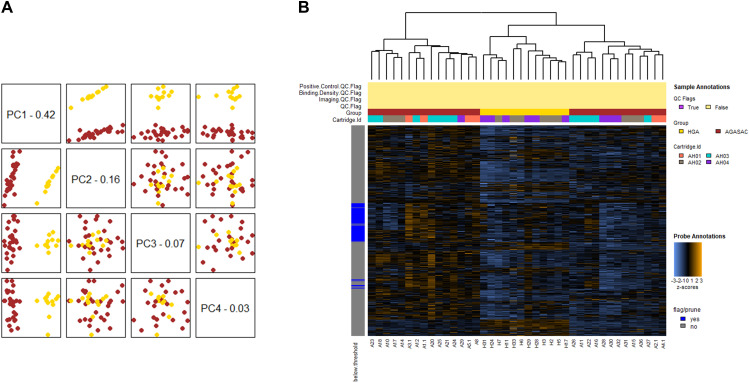
Gene expression data from nSolver^®^. **(A)** Principal component analysis (PCA) plot of the 40 primary tumour samples analysed with the nSolver^®^ Canine IO Panel. The HGA samples are represented by yellow dots, the AGASAC samples by red dots. **(B)** Heatmap of each sample’s full gene expression profile. H, hepatoid gland adenoma; A, apocrine gland anal sac adenocarcinoma; PC, principal component; QC, quality control; Id, identification.

Analysis again revealed a clear grouping of the two primary tumour sample groups in both the PCA plot and heatmap. The PCA plot showed a clear clustering of the two sample groups (Fig 4A), with most of the discrimination occurring along PC1. The eigenvalues of the first two PCs accounted for 58% of the variance in gene expression (PC1: 42%; PC2: 16%).

The heatmap of each sample’s gene expression profile likewise clearly separated the sample groups into three clusters (Fig 4B). Fifteen AGASAC samples clustered separately, while thirteen AGASAC samples clustered more closely with the twelve HGA samples.

**Fig 5:**
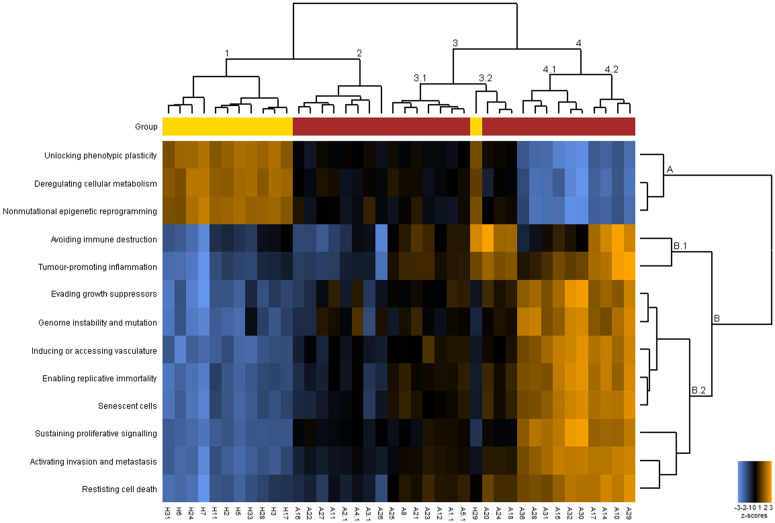
Heatmap of hallmark scores. The “pathway scoring module” in nSolver^TM^ generated this heatmap based on the data from the Canine IO Panel. The HGA samples are yellow, while the AGASAC samples are red. Blue, low scores; orange, high scores. Numbers in the dendrogram represent sample cluster identification, while letters denote hallmark clusters.

By using the “pathway scoring module” in nSolver^TM^ (see section 2.5), each sample’s gene expression profile linked to a hallmark of cancer was condensed into a single hallmark score. The heatmap of the hallmark scores presented a different clustering of the samples (Fig 5) compared to the heatmap of the gene expression profiles. Nevertheless, the two tumour types still formed distinct clusters, although one HGA sample (h29) clustered with AGASAC cluster 3.

All HGA samples exhibited high hallmark scores for “unlocking phenotypic plasticity”, “deregulating cellular energetics”, and “nonmutational epigenetic reprogramming” compared with the AGASAC samples (hallmark cluster A). In contrast, a subset of AGASAC samples (10 samples making up cluster 4) showed low hallmark scores for these three hallmarks. These AGASAC samples however displayed high hallmark scores for a set of hallmarks: “evading growth suppressors”, “genome instability and mutation”, “inducing or accessing vasculature”, “enabling replicative immortality”, “senescent cells”, “sustaining proliferative signalling”, “activation invasion and metastasis”, and “resisting cell death” (hallmark cluster B.2), while the HGA samples did not. The remaining AGASAC samples (clusters 2 and 3) did not exhibit as strong hallmark scores in hallmark clusters A and B.2.

Interestingly, we observed greater variability of hallmark scores for “avoiding immune destruction” and “tumour-promoting inflammation” (hallmark cluster B.1). Most HGA samples and eight AGASAC samples (cluster 1) had a low hallmark score for this cluster. The HGA sample h29, however, had a high hallmark score for this cluster, together with two AGASAC sample clusters (cluster 3.2 with 3 samples and cluster 4.2 with 4 samples). Histologically, all of these samples exhibited abundant stromal lymphofollicular aggregates. While these are a common feature in AGASACs [45], their presence in the HGA sample h29 indicates chronic inflammation.

**Fig 6:**
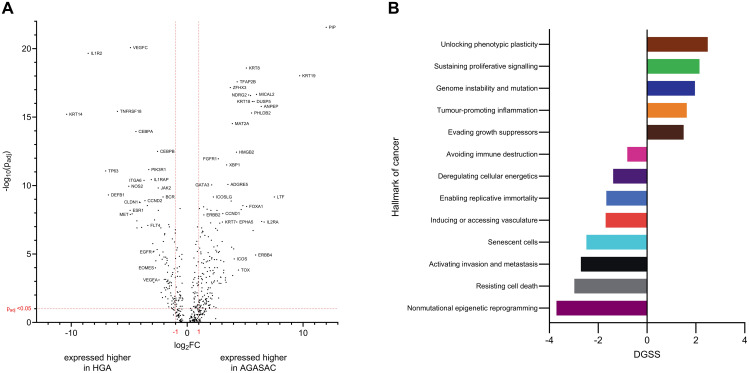
Differential gene expression data from nSolver^®^. **(A)** Volcano plot shows the differentially expressed genes calculated with nSolver^TM^ from the nCounter^®^ Canine IO Panel analysis. The apocrine gland anal sac adenocarcinoma (AGASAC) samples were compared to the hepatoid gland adenoma (HGA) samples. **(B)** The bar graph displays the directed global significance scores (DGSS) from the gene set analysis (GSA) module in nSolver^TM^ for the gene sets associated with thirteen hallmarks of cancer, comparing AGASAC primary tumour samples to the HGA baseline. The bar colours correspond to the colours used in Fig 1 of the most recent hallmarks of cancer publication [2]. FC, fold change.

In the DGE analysis, 239 of the 830 genes on the Canine IO Panel showed significant differential expression between AGASAC and HGA samples (S10 Table). Of these, 145 genes were expressed higher and 93 lower in the AGASAC samples. A volcano plot displays these DEGs (Fig 6A).

Furthermore, we compared the top 20 significantly DEGs from both methods and found a very high concordance in their expression direction (Table 2). Only one gene (CEBPB) exhibited an ambiguous expression direction, with a log_2_FC of 0 in the RNA-Seq data due to shrinkage performed in DESeq2. A very strong correlation based on the log_2_FC for all overlapping genes in both the nCounter^®^ Canine IO Panel and RNA-Seq was calculated (Pearson: 83.7%; Spearman: 74.5%). This correlation was stronger when applied to genes, which were significantly (p_adj_ < 0.05) differentially expressed in at least one method (Pearson: 85.5%; Spearman: 82.4%). A Jaccard coefficient of 58.3% for all overlapping significantly DEGs was calculated.

**Table 2:** Top 20 significantly differentially expressed genes (DEGs) common to both technologies.

Gene	RNA-Seq		nCounter^®^		Combined significance	Expression direction
	log_2_FC	p_adj_	log_2_FC	p_adj_		
TFAP2B*	3.9	1.37e-107	4.3	2.65e-18	108.3	consistent
PIP	13.6	2.49e-97	12	2.78e-22	99.0	consistent
KRT19*	11.2	1.52e-92	9.7	9.62e-19	93.6	consistent
IL1R2	−9.6	8.73e-79	−8.5	2.22e-20	80.5	consistent
MICAL2*	6.4	4.23e-71	6	2.20e-17	72.3	consistent
KRT8	13.3	6.25e-70	5.1	2.60e-19	71.7	consistent
MAT2A*	4.1	2.63e-70	3.9	3.01e-15	71.1	consistent
ST6GAL1	5.3	1.19e-67	5.5	2.69e-17	68.9	consistent
VEGFC	−4.8	4.50e-62	−4.9	8.49e-21	64.5	consistent
XBP1*	3.8	1.03e-60	3.4	3.12e-12	61.1	consistent
NDRG2*	5.6	5.28e-58	5.3	2.42e-17	59.6	consistent
ZFHX3*	3.8	1.68e-52	3.7	7.01e-18	54.5	consistent
PHLDB2*	5.5	1.24e-51	5.6	5.03e-16	53.2	consistent
ANPEP*	6.2	2.73e-50	6.4	1.68e-16	52.0	consistent
DUSP5*	6.2	2.19e-48	5.8	7.27e-17	50.3	consistent
KRT14	−11.1	1.63e-46	−10.4	6.26e-16	48.2	consistent
CEBPB	0	3.52e-45	−2.6	3.23e-13	46.2	ambiguous
TP63	−6.9	4.67e-44	−7	8.55e-12	44.7	consistent
CEBPA	−4.1	3.53e-39	−4.4	1.13e-14	40.9	consistent
ITGA6	−3.7	2.49e-38	−3.7	4.23e-11	39.0	consistent

Top 20 DEGs from both the nCounter^®^ Canine IO Panel with Panel Plus and RNA-Seq. The DEGs from nSolver^TM^ were merged and compared to those from DESeq2 with attention to the congruence of the log_2_FC direction. Ambiguous direction is identified via the rosé cell background. The combined significance was calculated based on the square root of sum of squares of -log_10_ of p-values. *, genes included on the Panel Plus; FC, fold change; p_adj_; adjusted p-value.

We then assessed the DEGs from the nCounter^®^ data within context of the hallmarks of cancer. Table 3 displays the number of DEGs per hallmark. Most absolute numbers were very similar between the higher and lower expressed group. However, two hallmarks showed a markedly different number of genes: “avoiding immune destruction” and “tumour-promoting inflammation”.

**Table 3:** Number of significantly differentially expressed genes per nCounter^®^ annotation based on the hallmarks of cancer.

Hallmark annotation (total number of genes)	Number of genes expressed higher in AGASACs than in HGAs	Number of genes expressed lower in AGASACs than in HGAs
Tumour-promoting inflammation (301)	68	35
Avoiding immune destruction (169)	35	17
Activating invasion and metastasis (151)	31	43
Evading growth suppressors (41)	10	5
Genome instability and mutation (42)	7	3
Deregulating cellular energetics (82)	9	14
Senescent cells (58)	11	7
Sustaining proliferative signalling (235)	50	43
Enabling replicative immortality (78)	14	11
Nonmutational epigenetic reprogramming (58)	11	9
Resisting cell death (114)	17	15
Unlocking phenotypic plasticity (119)	24	22
Inducing or accessing vasculature (65)	12	11

AGASACs, apocrine gland anal sac adenocarcinomas; HGAs, hepatoid gland adenomas.

We used the GSA module in nSolver^TM^ to further analyse the full differential expression analysis at the level of the hallmarks of cancer. The extent (strength and direction) of differential expression of each gene set was summarised using a directed global significance score (DGSS) from nSolver^TM^ (see section 2.5). The bar graph (Fig 6B) displays the DGSSs for the gene sets associated with thirteen hallmarks of cancer in the AGASAC primary tumours compared to the HGA baseline. No p-values are provided with these scores; thus, the statistical power is limited. Yet, the scores still can give an impression of differential expression of gene sets (see section 2.5), in this study’s context of the hallmarks of cancer.

Five hallmarks were scored higher in the AGASAC samples: “unlocking phenotypic plasticity” (2.49), “sustaining proliferative signalling” (2.15), “genome instability and mutation” (1.96), “tumour-promoting inflammation” (1.63), and “evading growth suppressors” (1.5). In contrast, eight hallmarks were scored higher in the HGA samples: “nonmutational epigenetic reprogramming” (−3.71), “resisting cell death” (−2.98), “activating invasion and metastasis” (−2.71), “senescent cells” (−2.47), “inducing or accessing vasculature” (−1.7), “enabling replicative immortality” (−1.67), “deregulating cellular energetics” (−1.39), and “avoiding immune destruction” (−0.82).

### Differences in particular genes of interest seen in tumour immune landscape, proliferation, and angiogenesis

We selected genes of interest to elucidate whether differential gene expression was evident in genes which are crucial and familiar in the context of cancer biology. Individual genes were chosen based on their key representative roles in the hallmarks of cancer with the full knowledge that this was not an exhaustive list and that single genes are often involved in multiple hallmarks. The full list can be found in S11 Table. As the Canine IO Panel with Panel Plus only included 820 genes, many genes of interest could only be found in the RNA-Seq data. Some of these findings are further discussed below.

### A comparison between AGASAC primary tumours and their lymph node metastases reveals little transcriptomic differences

Lastly, we evaluated transcriptomic differences between AGASAC primary tumours and their lymph node macrometastases by conducting a separate analysis of the RNA-Seq and nCounter^®^ data. We applied DESeq2 with a paired design model correcting for patient specific effects on the gene expression data (intra-patient correction) and compared the four AGASAC primary tumours (a.1.1, a2.1, a4.1, a5.1) with their corresponding lymph node metastases (a.1.2, a2.2, a4.2, a5.2) analysed with RNA-Seq (S12 Table). This analysis identified only six significantly DEGs that were higher expressed in the primary tumours: ACTA1, NEB, OGN, POSTN, MYL11, and SLN. In contrast, one DEG (IGF2) was higher expressed in the metastases.

We separately analysed the five sample pairs analysed with nCounter^®^ – including the same samples as above plus a3.1 and a3.2 – using nSolver^TM^. Unlike DESeq2, the nSolver^TM^ analysis did not identify any significantly DEGs (S13 Table).

## Discussion

The transcriptomic profiles of the two most common, yet biologically very different canine perianal tumours revealed differences in several aspects and genes associated with the hallmarks of cancer. Overall, these two tumours could clearly be distinguished from one another in several aspects, which reflect clinical and microscopic observations. Data analyses revealed that this was not only due to their different biological behaviours, but also likely due to non-tumour related characteristics such as the cell types of origin and their modes of secretion.

Interestingly, very close clustering of the HGA samples was revealed, indicating very similar transcriptional processes among the individual tumours tested. In this manner, the HGAs seemed more homogenous than the AGASACs.

On the other hand, other transcriptomic differences were not as strong as expected for two tumour entities with vastly distinct clinical and metastatic behaviours. Surprisingly, neither the gene expression data nor the DGE analyses clearly revealed an over-representation of the “activating invasion and metastasis” hallmark or of particular genes typically linked to metastasis in the AGASAC gene set. Thus, a general transcriptomic separation between benign and malignant behaviour seemed not as clear-cut in this particular comparison and warrants a closer look at specific gene sets related to the hallmarks of cancer and individual genes of interest.

### Over-representation and gene set enrichment analyses point toward strong differences in cell metabolism in the HGA, while the AGASAC displays enrichment of immune-related terms

Surprisingly, no hallmarks were significantly over-represented in the AGASAC gene set. Stronger distributions of gene sets per hallmark may allude to underlying biological differences in “sustaining proliferative signalling”, “replicative immortality”, “evading immune evasion”, “tumour-promoting inflammation”, and “tissue invasion and metastasis”. These may have become more apparent if the gene list used as a comparison would have been based on canine cancer studies, which are lacking on a molecular level, or with a larger sample size.

In the HGA gene set, only “reprogramming energy metabolism” was significantly over-represented. This also fits well to the MSigDB results. Stronger distributions of gene sets per hallmark may allude to underlying biological differences in “resisting cell death” and “sustaining angiogenesis”.

Gene set enrichment analysis on the basis of the MSigDB hallmark gene sets, however, revealed more significant differences in the transcriptomic landscapes of the compared tumours. The AGASAC gene set exhibited a strong enrichment of immune-related pathways indicative of activation of innate and adaptive immune responses, as well as inflammatory and proliferative signalling. Specifically, activation of IFN-γ (type II) and IFA-α (type I) interferon responses are associated with increased antigen presentation (via MHC-I), immunosurveillance, and recruitment of cytotoxic lymphocytes [49]. Similarly, TNF-α and IL6/JAK/STAT signalling indicate inflammatory and pro-survival (immune escape) pathways, which are implicated in cancer-associated chronic inflammation, immune cell infiltration, and potential resistance to apoptosis [50]. NF-κB activation is typical of inflammatory carcinomas, where it promotes tumour survival, EMT, and resistance to apoptosis [51]. In the context of the AGASAC, these data point toward a “hot” tumour immune phenotype with enriched terms compatible with pro-inflammatory signalling, immune infiltration, and chronic inflammation. In many epithelial cancers, a strong interferon signature suggests ongoing immune recognition [52]. The enrichment of cell cycle regulation-related terms (G2/M checkpoint, E2F targets) imply proliferative capacity, cell cycle dysregulation with loss of checkpoint control and E2F-driven progression and genomic instability, underscoring AGASAC’s malignant character. Additionally, the enrichment of androgen and oestrogen response terms suggest some degree of hormone receptor activity. The enrichment of apoptosis may indicate stress-induced apoptotic pressure, likely due to immune activation.

The HGA gene set was dominated by terms related to metabolic specialization and differentiation. These included adipogenesis, oxidative phosphorylation, cholesterol homeostasis, fatty acid metabolism, and peroxisome, indicating a more differentiated, less malignant phenotype. mTORC1 signalling, p53 pathway, and glycolysis point towards more nutrient-sensing, cellular stress responses, lipid biosynthesis, and energetic adaptation, which support cell growth. These enriched terms fit to the well-differentiated, metabolically active HGA with limited immune activation, typical of “cold” tumours. Specifically, these terms suit the lipogenic, sebaceous/holocrine secretory nature of the hepatoid cells. The oestrogen response terms are enriched stronger in the HGA gene set, reflecting the suggested hormone-dependant physiology of perianal glands and tumours [53,54]. Enrichment of the p53 pathway suggests an intact cell-cycle restraint. Finally, glycolysis, ROS pathway, and hypoxia reflect an increased metabolic flexibility and hypoxic stress. Together, these terms suggest that HGAs heavily rely on altered lipid synthesis, as well as oxidative phosphorylation to meet their energetic and biosynthetic demands for proliferation. However, the observed enrichment of lipid metabolism pathways could also be supported by the holocrine secretion mode of hepatoid glands, which involves the release of lipid-rich cellular contents upon cell lysis. This intrinsic physiological characteristic of the original tissue may predispose these tumours to leverage lipid metabolic pathways for growth and survival, potentially explaining the revealed distinct metabolic signature.

Thus, gene set enrichment analyses revealed distinct transcriptomic landscapes in AGASACs and HGAs, strongly suggesting fundamentally different underlying biological mechanisms driving these two canine tumours.

However, some challenges to this transcriptomic picture were brought forth by the DGSS from nSolver^TM^. Here, the hallmarks “activating invasion and metastasis” and “avoiding immune destruction” were scored in favour of the HGA samples. The surprisingly higher DGSS for “activating invasion and metastasis” clearly contradicts the opposite biological behaviour commonly observed in this entity. This observation is likely due to the fact that the nSolver^®^ gene panel only included 820 genes, compared to 27,493 captured with RNA-Seq. A bias based on the genes represented on the panel is probable, which likely skewed the picture.

Additionally, the discrepancies are possibly due to the fact that the annotation file provided by NanoString is not in itself an ontological database, only based on these. However, these databases are subject to updates and have a human focus. In this context it is important to note that orthologous genes do not always retain identical function across species. A gene may play a specific role in humans that differs in dogs [55]. Such divergence can complicate the interpretation of enrichment results. This highlights the need to carefully consider species-specific gene functions when drawing biological conclusions from cross-species analyses. Other hallmarks are also far better understood on a molecular level and conserved between species, i.e., “sustaining proliferative signalling” in comparison to others, such as “activating invasion and metastasis”.

### Potential implications of the top 20 differentially expressed genes

We examined the top 20 DEGs from RNA-Seq in the context of their potential roles in tumour progression. Newly observed in the AGASAC is the high expression of ARHGEF40, which promotes malignant behaviour through RhoA-mediated activation of the AKT-Wnt/β-catenin pathway by enhancing proliferation and invasion. It is also associated with lymph node metastasis [56]. This many underpin AGASAC’s invasiveness and lymphatic spread. TFAP2B over-expression similarly points to enhanced angiogenesis, as it inhibits apoptosis through modulating ERK/p38, MAPK, and VEGF pathways [57]. Beyond confirming epithelial differentiation, elevated KRT19 and KRT8 expression extend previous findings [58,59] by indicating their potential diagnostic utility for AGASACs. For the first time in this context, SEMA3C and MICAL2 appear as candidate mediators of tumour angiogenesis [60] and actin cytoskeleton dynamics with an effect on epithelial-mesenchymal transition (EMT) and invasion [61,62], respectively. This may imply coordinated regulation of invasion and vascular remodelling. In contrast, high expression of FRZB, a Wnt antagonist related to decreased invasiveness and inhibition of Met signalling [63], and NDRG2, a metastasis suppressor [64], are both associated with reduced tumour aggressiveness in other cancers. This finding suggests distinct regulatory dynamics in AGASAC biology regarding these two genes. Additionally, high expression of MAT2A, which is linked to redox balance in proliferating cells [65], and XBP1, which mediates unfolded protein response [66], highlights adaptation to metabolic and stress responses. ST6GAL1 and FUT2 alter cell surface glycosylation patterns, potentially affecting cell adhesion, metastasis, or immune evasion. GCGC encodes for the glucagon receptor with an unclear oncogenic role in AGASACs. Summarizing, these findings suggest AGASAC progression involves Wnt-AKT activation (via ARHGEF40), TFAP2B, and SEMA3C-driven angiogenesis, as well as MICAL2-mediated invasion.

Among the genes lowly expressed in the AGASACs but higher expressed in the HGAs, ARHGAP25 is notable as it negatively regulates cell migration and invasion. It is down-regulated in osteosarcoma and other solid tumours [67]; lower expression in the AGASACs aligns well to this observation. SCARB1 is involved in lipid metabolism [68]. Together with CIDEA, likewise associated with lipid droplet regulation [69], both are likely associated with the holocrine secretion mode of HGAs. Interestingly, our data newly suggest that differential expression of both IL1R2 and VEGFC may mark distinct angiogenic programmes in this tumour. HGAs seem to retain a more IL1R2/VEGFC-driven vascular signature. Mechanistically, intracellular IL1R2 complexes with c-Fos to upregulate IL6 and VEGFA, thus promoting endothelial cell proliferation [70]. VEGFC also correlates with tumour vascularization. Lower DSG1 expression, together with reduced KRT79, supports loss of epithelial cell adhesion, compatible with EMT in AGASACs. ZNF365 is involved in DNA repair and genome stability [71]. TRIM29 has a role in ubiquitination signalling [72]. With context-dependent roles in cancer, following genes can act both anti- or pro-tumourigenic: CLMP functions as a tumour suppressor in colorectal cancer [73]. SPINK2 inhibits EMT, cell migration and invasion, urokinase-type plasminogen activator signalling [74]. Low SPINK2 expression correlates with aggressive disease in testicular cancer [75]. LGALS7B has a role in cell adhesion, protease inhibition, and immune regulation [76]. Collectively, these patterns emphasize the AGASACs diverge from HGA by attenuating multiple adhesion, lipid metabolism, and putative tumour-suppressor pathways, keeping in line with their more aggressive clinical behaviour.

### Discriminating immune landscape and metastasis-associated gene expression is evident in the AGASAC

Previous immunohistochemical studies have shown that AGASACs express PD-L1 and intra-tumour as well as peri-tumour CD3+ lymphocytes express PD-1 with some variability [28]. Corresponding data has demonstrated that PD-L1 expression negatively affected survival of dogs treated solely with surgery. PD-L1 expression has been shown in 95% of examined AGASACs. Pilot studies using monoclonal antibodies targeting PD-1 in a dog with oral adenocarcinoma [77] and PD-L1 in dogs with oral melanoma or undifferentiated sarcomas [78,79] have demonstrated promising clinical regression. In our data, PDCD1 (PD-1) was significantly differently expressed, while CD274 (PD-L1) was not. However, other genes involved in T-cell exhaustion were expressed higher in the AGASAC samples as well, namely TOX, LAG3, IDO1, CYBB (NOX2), CTLA4, HAVCR2 (TIM-3), ENTPD1, and ADORA2A. Thus, AGASAC seems like a promising tumour to investigate treatment with immune checkpoint inhibitors.

Further thought should be given to trying to establish an “Immunoscore” for inflamed tumours in veterinary medicine [80–82]. This could be linked to prognosis and treatment options for different canine tumours. The AGASAC is a potential candidate for characterising the *in-situ* cell infiltrate, as there is enrichment of pathways related to antigen presentation. However, the AGASAC samples did not homogenously exhibit the same degree of immune response, as established by the subgrouping in the nCounter^®^ data analysis. Specifically, the lymphocyte populations infiltrating the core of the tumour and the invasive margin of AGASACs should be investigated separately, including type, functional orientation, location, and density. A previous study had characterised the density and distribution of immune cell infiltrates [83] and found that T-cells and macrophages were the most abundant. Tumours with metastasis had significantly higher numbers of macrophages, while those without metastasis had significantly higher numbers of T-cells. This suggests that increased T-cell infiltration may be protective against metastasis and tumour progression, whereas increased macrophage infiltration may promote it.

In this context, some AGASACs exhibited features compatible with the “hot” or “immune inflamed” immune contexture, while the HGA is consistent with “cold” or “immune desert” landscape on the tumour immunity continuum [80]. The AGASACs showed increased expression of genes related to antigen presentation, such as the canine MHC-I and -II genes. Additionally, genes indicative of cytotoxic activity, such as CTSW, GZMA, GZMB, and CD8B were higher expressed. Conversely, the HGAs exhibited higher expression of the immunosuppressive gene NT5E (CD73), as well as genes related to hypoxia, such as HIF1A, NOS2, NOS3, VEGFs, and PDGFs and enriched fatty acid metabolism pathways, as well as low MHC-I.

Among the two compared tumours, only the AGASAC metastasises readily and early to the locoregional lymph nodes [84]. Identifying specific genes that initiate metastasis has however proven difficult to impossible, as a complex evolution with diverse processes lead to dissemination. Oncogenic pathways that drive tumour metastasis are often associated with acquiring a stemness phenotype [85]. Three well-known, metastasis-associated genes however were differentially expressed in this tumour comparison, with EPCAM and CD24 higher expressed in the AGASAC samples and CD44 higher expressed in the HGA samples. EPCAM is often highly expressed in epithelial cancers and their metastases, and serves as a prognostic marker. It is involved in the regulation of cell adhesion, proliferation, migration, stemness, and EMT of carcinoma cells [86,87]. Fittingly, EPCAM was expressed higher in the AGASAC samples and may thus be considered a biomarker for this tumour in dogs. The relationship of expression between the adhesion molecules CD44 and CD24 has been intensely researched, with contradicting data on prognostic significance and influence on gaining stemness and metastatic potential. A CD44^+^/CD24^–^ phenotype is generally linked to cancer stem-like cells, associated with high grade and lymph node infiltration, however in canine mammary tumours the opposite has been shown. In canine mammary tumours, the CD44^+^/CD24^–^ phenotype was associated with a better overall survival and the CD44^–^/CD24^+^ and CD24^+^ phenotype was associated with poor prognosis, high grade, and metastasis [88]. In our tumour comparison, CD44 was expressed higher in the benign HGA and CD24 was expressed higher in the malignant AGASAC, consistent with the observation that CD44-low/CD24-high tumours have a poorer prognosis in dogs.

### Little difference between the AGASAC primary tumours and their nodal metastases

The comparison between AGASAC primary tumours and their lymph node metastases revealed a pair-wise close clustering of all primary tumours with their corresponding metastasis, indicating little transcriptomic differences. This was further substantiated by the presence of only six higher expressed genes in the primary tumour samples analysed with RNA-Seq. These included ACTA1, NEB, OGN, POSTN, MYLPF, and SLN. POSTN especially stands out, as it is involved in supporting motility and migration of epithelial cells, invasion, proliferation, and EMT. Over-expression is reported in several types of cancer, most frequently in the stroma or extracellular matrix of tumours and is linked to poor prognosis [89–92]. Thus, POSTN might be involved in metastatic dissemination of AGASAC cells. OGN has been implicated in diverse, often contradicting functions associated with neoplasia. Studies implicate OGN with anti-tumour effects, such as inhibition of cell proliferation and invasiveness, as well as reversal of EMT, while others report a correlation with EMT signature and poor prognosis and tumour promoting effects [93–95]. The other four genes are related to skeletal muscle cells, but we can exclude significant muscle contamination in our samples based on our histological observations. IGF2 was found higher expressed in the nodal metastasis samples. Higher expression of IGF2 has been linked to metastatic progression and immune evasion. IGF2 helps establish a supportive stromal and vascular environment in lymph nodes. Specifically, IGF2 reprograms distant sites by recruiting immunosuppressive myeloid cells and remodelling the extracellular matrix, creating a favourable environment for circulating tumour cells, creating a pre-metastatic niche [96]. IGF2 also activates the CXCL5/CXCR2 axis**,** thereby attracting cancer cells to metastatic sites [97]. CXCR2 however was not differentially expressed and CXCL5 is not annotated in the canine genome. M2-like tumour-associated macrophages [96] and cancer-associated fibroblasts [98] in the tumour microenvironment (TME) secrete IGF2, which suppress cytotoxic T-cell activity. This aligns with the observation that metastatic AGASACs show lower T-cell and higher macrophage infiltration, a pattern linked to IGF2-driven immune evasion [83]. Interestingly, a previous study comparing the gene expression landscape of primary AGASACs that did or did not metastasise failed to identify a subset of the genes that could predict metastatic behaviour [99].

Surprisingly, terms related to angiogenesis were not clearly enriched in either of the two tumour entities. However, angiogenesis-specific genes of interest, especially the VEGF and VEGFR genes, were expressed higher in the HGA samples (S11 Table) and a trend was visible in the HGA gene set (Fig 4B). Associating this information with the histologic characteristics of these tumours reveals some interesting observations. Necrosis is seldomly seen in HGAs, even at larger tumour sizes. Additionally, the interlobular stroma is usually rich in blood vessels [84]. Conversely, necrosis is much more common in AGASACs and lymphatic metastatic spread is far more frequent than the haematogenous route [8,10]. This might explain the preponderance of angiogenic factors in the HGA samples. Furthermore, HIF1A, NOS2, and NOS3 were higher expressed in the HGA samples, indicating that mechanisms against hypoxia are activated in the HGA.

### Few genes could be used for diagnostic purposes

A small selection of genes can likely diagnostically differentiate these two entities and could be used in a clinical or pathologic bench-top test. Prolactin-inducible protein (PIP) was one of the most DEGs and high expression was found in the AGASACs, with only few reads in the HGAs (Table 1). PIP can seemingly be used alone to differentiate AGASAC from HGA. As a marker for apocrine secretion [100], PIP is seemingly not lost in malignant transformation of the apocrine epithelium of the canine anal sac and is also present in nodal metastases. Interestingly, PIP has been proposed as a biomarker for nodal micrometastases in human breast cancer [101], linked to immune regulation and cell cycle progression [102]. Specifically, PIP is required for the progression through the G1 phase, mitosis, and cytokinesis in cells of three breast cancer subtypes with an enrichment of genes associated with cell cycle genes, such as CCND1, CCNB1, BUB1, and FOXM1 [102]. CCND1 was also expressed higher in the AGASAC samples, together with CDK1 and CDK3. Although higher PIP expression has been associated with better prognosis during early breast cancer development, its role in enhancing metastatic spread seems paradoxical [103,104]. Interestingly, PIP exerts an immunoregulatory role in immune response-evoking breast cancers [105]. PIP-expressing breast tumours exhibit higher percentages of NK cells and reduced numbers of Th2 cells in the tumour environment [104]. Additionally, PIP is a regulator of CD4+ Th1-cell-mediated immunity with pro-inflammatory cytokine production [106,107]. Infiltrating lymphocytes, both in the stroma and between the tumour cells is a common finding both in AGASACs and some forms of human breast cancer, making PIP an interesting candidate for further investigations.

### Consideration of methodological aspects and limitations

Both methods clearly separated the transcriptomic landscapes of the two tumours with overall similar results of DGE. However, the data on enriched gene sets differed in certain aspects. Especially the focussed approach on specific genes with the nCounter^®^ panel may have skewed the picture through a limited perspective of only 820 pre-selected genes.

As a second methodological limitation, the innate biological differences of the cells (i.e., the apocrine versus holocrine modes of secretion), may have obscured the elucidation of specific processes such as immuno-oncologic activity and energy metabolism.

Another restriction to our study relates to the aspect of inter- and intra-tumour heterogeneity [108,109], as whole cross sections of entire solid tumours or segments thereof were utilised for RNA extraction. Thus, RNA from different tumour cell subpopulations and cells of the TME were analysed in bulk (bulk sequencing). Regionally different transcriptional profiles within the same tumour could thus not be identified in this approach. Tumour heterogeneity may therefore have caused some of the unclear or surprising results of our study.

Unfortunately, no rigorous medical history or clinical follow-up data were available for most of the employed samples and despite trying to gather data on survival time or recurrence, most clinicians we contacted were unable to offer such information. Thus, our gene signatures or groupings could not be correlated with clinical profiles of individual tumours in meaningful numbers.

Finally, further restrictions to our study relate to bioinformatic and gene set enrichment aspects. Although the canine genome is overall well annotated, some gene annotations are still incomplete, as we found for the glucose transporter (GLUT1–4), death receptors, and TRAIL receptor genes. This incompleteness stems mainly from the fact that despite recent advances and improved reference genome assemblies, functional annotations are lacking or under-characterised. Annotation efforts are ongoing, with large-scale projects working to fill gaps by integrating transcriptomic and epigenomic data across diverse tissues and breeds. Until these efforts achieve more comprehensive coverage, gaps in gene annotation can impact the interpretation of gene function in disease and cancer context [110]. Certain pathways and genes of interest could thus likely not be fully considered and were missing for the gene set enrichment analyses. Further, the gene annotation sources used for the nSolver^TM^ annotation file, such as KEGG, GO, and Reactome, are based on human or murine genetics and may not be fully transferable to the dog [111,112]. The same is true about the gene list compiled for cancerhallmarks.com [46].

## Conclusion

In summary, our transcriptomic landscaping of two clinically highly contrasting canine tumours of unlike cells of origin revealed differences relating to the hallmarks of cancer. These mirrored both discrepancies in their biological behaviours as well as cell types of origin. However, benign versus malignant tumour behaviour and even more specific properties such as invasion and metastasis and angiogenesis could not be recapitulated and the over-expression analysis did not display as strong differences as expected. Some of our findings, particularly those with therapeutic potential, should be followed up using approaches with more spatial or cell-type-specific resolution, such as spatial omics or scRNA-Seq, optimally accompanied by more clinical patient data. To improve the robustness and reproducibility of our findings, the establishment of veterinary cancer biobanks that curate well-annotated, high-quality samples linked to detailed clinical metadata are crucial. This need should be reiterated, despite the numerous challenges involved. Similar to initiatives like The Cancer Genome Atlas (TCGA) in human oncology, organising these resources into lager and separate cohorts for learning, testing, and validation could significantly enhance the identification of consistent molecular signatures and biomarkers in veterinary cancers, especially the AGASAC.

## Supporting information

S1 Table Tissue sample information including Gene Expression Omnibus (GEO) accessions. (XLSX)

S2 Table RNA quality data. (XLSX)

S3 Table Key parameters used in the transcriptome analysis software. (XLSX)

S4 Table List of Panel Plus gene probes. (XLSX)

S5 Table Annotation file based on the hallmarks of cancer for the nSolver^TM^ 4.0 with Advanced Analysis 2.0 software used in this study with the nCounter^®^ Canine IO Panel with a 30 gene Panel Plus. (CSV)

S6 Table Mapping of hallmarks of cancer annotations to whole transcriptome analysis (WTA) annotations by NanoString’s gene curation team. (XLSX)

S7 Table Differential gene expression (DGE) data from DESeq2 for the comparison AGASAC primary tumours versus HGAs. (XLSX)

S8 Table Data from the over-representation analysis based on the hallmarks of cancer from cancerhallmarks.com. (XLSX)

S9 Table Data from the gene set enrichment analysis based on the MSigDB hallmarks gene set from cancerhallmarks.com. (XLSX)

S10 Table Differential gene expression (DGE) data from nSolver^TM^ 4.0 with Advanced Analysis 2.0 for the comparison AGASAC primary tumours versus HGAs. (XLSX)

S11 Table List of genes of interest separated by significant differential expression in either the data from RNA-Seq or nCounter^®^, or from both methods. (DOCX)

S12 Table Differential expression analysis data from DESeq2 for the comparison AGASAC primary tumours versus nodal metastases. (XLSX)

S13 Table Differential gene expression (DGE) data from the nSolver^TM^ 4.0 with Advanced Analysis 2.0 for the comparison AGASAC primary tumours versus nodal metastases. (XLSX)
