# Supplementary material for: Extremes on the benign-malignant tumour spectrum: Distinct transcriptomic landscapes between two common canine perianal neoplasms based on the hallmarks of cancer
Source: PLoS One. 2026 Jun 22;21(6):e0351773. doi: 10.1371/journal.pone.0351773 (PMC13286202; doi:10.1371/journal.pone.0351773)
Supplement: S11 Table — (DOCX) [file pone.0351773.s011.docx]

**Table S11**. **Genes of interest (GOIs) in the context of the hallmarks of cancer.**

GOIs are separated based on their significant (p_adj_ < 0.05) differential gene expression (DGE) either in the hepatoid gland adenoma (HGA) with a log_2_FC < -1 or in the apocrine gland anal sac adenocarcinoma (AGASAC) primary tumours with a log_2_FC > 1 and in which method (both, RNA-Seq, or nCounter^®^) this was evident in.

|  | **DGE in both methods** | | **DGE in RNA-Seq** | | **DGE in nCounter^®^** | | **No significant DGE** |
| --- | --- | --- | --- | --- | --- | --- | --- |
|  | **for HGA** | **for AGASAC** | **for HGA** | **for AGASAC** | **for HGA** | **for AGASAC** |  |
| **Tumour-promoting inflammation** | | | | | | | |
| NF-κB transcription | NFKBIA |  |  |  |  |  | NFKB1, NFKB2, RELA, RELB, REL |
| JAK-STAT signalling | JAK2, STAT5A | STAT1, STAT2 |  |  |  |  | JAK1, JAK3, TYK2, STAT3, STAT4, STAT5B, STAT6 |
| inflammasome |  |  |  |  |  |  | NLRP1, NLRP3, NLRP10, NLRP12, NLRC4, MEFV, CARD, PYCARD (ASC) |
| co-stimulatory signalling | TNFRSF18 (GITR) | ICOS, ICOSLG (B7-H2), TNFRSF14 (HVEM) |  |  | CD80 (B7-1) | IL2RB (CD122), CD28 | CD27, CD86 (B7-2), CD40, CD40LG, TNFRSF4 (OX40), TNFSF4 (OX40L), TNFRSF9 (CD137), TNFSF9 (CD137L) |
| MHC I |  | DLA-79, DLA-64, DLA88 |  |  |  |  |  |
| MHC II |  | DLA-DMB, DLA-DQA1, DLA-DQB1 |  |  |  | DLA-DOB, DLA-DMA | DLA-DOA |
| T-cells, incl. T-cell receptor (TCR) protein complex |  | CD3E (ε), CD3G (γ) |  |  |  |  | CD3D (δ), CD247 (ζ), CD4, CD6, TRAT1, TBX21 |
| regulatory T-cells (T_regs_) |  | IL2RA (CD25) |  |  |  |  | FOXP3 |
| cytotoxic T-cells or natural killer (NK) cells |  |  |  |  |  | CD8B, CTSW, GZMA, GZMB | CD8A, PRF1, KLRD1, KLRB1, KLRK1, NKG7 |
| macrophages |  |  |  |  |  |  | AIF1 (IBA-1), CD68, CD163, CD84, ADGRE1 |
| mast cells |  |  |  |  |  |  | CPA3, HDC, MS4A2 |
| neutrophils |  |  |  |  |  |  | MPO, ELANE, CSF3R, S100A12 |
| B-cells |  |  |  |  |  |  | PAX5, CD19, FCRL2, TNFRSF17, MS4A1 (CD20) |
| cytokines & receptors | IL1R2, IL17RA | TGFB1, IL15, CCL3, CCL4, CCL5, CXCL10, CXCL16, CCR5, IL2RA, IL6R, IL11RA, IL13RA1, IL21R, IFNGR1 | IL10RB, IL17RC | CSF1 (M-CSF) | IL1A, IL2RB, IL17A, IL4R, CXCL12, CXCL14 | IL1B, IL12B, CXCR6, TNF, IL13RA2, CCL2 (MCP-1) | IFNG (IFN-γ), TGFB2, IL2, IL5, IL6, IL7, IL10, IL11, IL12A, IL17B, IL17D, IL18, IL20, IL21, IL23A, IL27, IL33, IL37, CSF2 (GM-CSF), CCR1, CCR2, CCR3, CCR4, CCR6, CCR7, CCR8, CCR9, XCR1, CX3CR1, CXCL13, CXCL8, CXCL11, CXCL17, CXCR1, CXCR2, CXCR3, CXCR4, CXCR5, IL1R1, IL3RA, IL7R, IL10RA, IL12RB1, IL12RB2, IL15RA, IL17RE, IL17RB, IL18R1, IL20RA, IL20RB, IL22RA2, IL22RA1, IL23R, IL27RA, IL31RA, CCL1, MIF |
| prostaglandin action |  | PTGER4 (EP_4_) |  | PTGES |  |  | PTGS1 (COX-1), PTGS2 (COX-2), PTGER1 (EP_1_), PTGER2 (EP_2_), PTGER3 (EP_3_), PTGES2, PTGES3, HPGDS (PDGS), PTGDR (DP_1_), PTGDR2 (DP_2_), HPGD (15-PGDH) |
| **Avoiding immune destruction** | | | | | | | |
| inhibitory immune checkpoint molecules, T-cell exhaustion | TCF7 (TCF-1), NT5E (CD73) | PDCD1 (PD-1), LAG3 |  | CYBB (NOX2) | VSIR (VISTA), EOMES | CTLA4, ADORA2A HAVCR2 (TIM-3), ENTPD1, TOX, IDO1 | CD274 (PD-L1), ADORA2B, CD244, CD276 (B7-H3), VTCN1 (B7-H4), BTLA, TIGIT |
| **Inducing or accessing vasculature** | | | | | | | |
| anti-angiogenic factors |  |  |  |  |  |  | THBS1 (TSP1), CD36, TNFSF15 (VEGI), IFNB1 |
| vascular endothelial growth factors & receptors | VEGFC |  | VEGFB |  | VEGFA, KDR (VEGFR-2), FLT4 (VEGFR-3) |  | VEGFD, FLT1 (VEGFR-1) |
| angiopoietins & receptors |  |  | TIE1, ANGPTL4 | ANGPTL2 | ANGPT1, ANGPT2 |  | TEK (TIE2), ANGPT4, ANGPTL1, ANGPTL3, ANGPTL6, ANGPTL7 |
| platelet-derived growth factors & receptors | PDGFA | PDGFC | PDGFD |  |  |  | PDGFB |
| fibroblast-growth factors & receptors |  | FGFR1 (FLT2) |  | FGFRL1, FGF20 |  |  | FGFR2, FGFR3, FGFR4, FGF1, FGF2 (bFGF), FGF4, FGF5, FGF7, FGF8, FGF9, FGF10, FGF11, FGF12, FGF13, FGF14, FGF16, FGF18, FGF19, FGF21, FGF22, FGF23 |
| nitric oxide syntheses | NOS2 (iNOS) |  | NOS3 (eNOS) |  |  |  |  |
| ephrins & Eph receptors |  | EPHA5 | EFNB1, EFNB2 | EPHA1, EPHA6, EPHB3, EFNB3 |  |  | EPHA2, EPHA3, EPHA4, EPHA7, EPHA8, EFNA1, EFNA2, EFNA3, EFNA4, EFNA5, EPHB1, EPHB2, EPHB4, EPHB6 |
| **Activating invasion and metastasis** | | | | | | | |
| epithelial-mesenchymal transition (EMT) |  |  | CTNNA1 | CTNNA3 |  |  | CDH1 (E-cadherin), CDH2 (N-cadherin), SNAI1 (SNAIL), SNAI2 (SLUG), ZEB2, CTNNA2, CTNNB1 (β-catenin), CTNND1 (p120) |
| cell adhesion molecules (CAMs) |  | EPCAM | CDH15, DSC1, DSC3, DSG1, DSG3, DSG4 | CDH20, CD24 | CD44, PECAM1 |  | CDH3, CDH4, CDH5, CDH6, CDH7, CDH8, CDH10, CDH11, CDH12, CDH13, CDH17, CDH18, CDH19, CDH22, CDH23, CDH24, DSC2, DSG2, SELE, SELL, SELP, SELPLG, NCAM1, ICAM1, VCAM1, MADCAM1 |
| keratins | KRT14 | KRT7, KRT8, KRT18, KRT19 | KRT16 | KRT23 | KRT79 |  | KRT1, KRT2, KRT3, KRT4, KRT5, KRT6A, KRT9, KRT10, KRT13, KRT15, KRT17, KRT20, KRT25, KRT27, KRT28, KRT31, KRT32, KRT33A, KRT33B, KRT34, KRT35, KRT36, KRT38, KRT40, KRT42, KRT49, KRT71, KRT72, KRT73, KRT74, KRT75, KRT77, KRT78, KRT80, KRT81, KRT82, KRT83, KRT85, KRT86, KRT88, KRT89, KRT124, KRT222 |
| integrins | ITGA6 (CD49f) | ITGAM (CD11b) | ITGB4 (CD104), ITGB5 | ITGA8 |  | ITGAX (CD11c) | ITGAE (CD103), ITGAD (CD11d), ITGAL (CD11a), ITGAV (CD51), ITGA1 (CD49a), ITGA2, ITGA2B, ITGA3, ITGA4 (CD49d), ITGA5 (CD49e), ITGA7, ITGA9, ITGA10, ITGB1 (CD29), ITGB2 (CD18), ITGB3 (CD61), ITGB6, ITGB7, ITGB8 |
| tight junctions |  |  |  | CGN, CLDN3, CLDN7, CLDN18 | CLDN1 |  | F11R (JAM1), JAM3, OCLN, CLDN4, CLDN5, CLDN6, CLDN8, CLDN9, CLDN10, CLDN11, CLDN12, CLDN14, CLDN15, CLDN16, CLDN17, CLDN19 |
| matrix metalloproteinases (MMPs) |  |  | MMP14 | MMP7 |  | MMP9 | MMP1, MMP3, MMP8, MMP11, MMP13, MMP19 |
| tissue inhibitors of metalloproteinases (TIMPs) |  |  |  | TIMP1, TIMP2 | *not included on panel* | | TIMP3, TIMP4 |
| metastasis suppressors |  |  | DLC1 |  | *not included on panel* | | NME1 (NM23), CD82 (KAI1), BRMS1, KISS1, MED23, GAS1, PEBP1 (RKIP), CAVIN2 (SDPR) |
| **Enabling replicative immortality** | | | | | | | |
| telomerase |  |  |  |  |  |  | TERT, TEP1, DKC1 |
| telomere length maintenance |  |  |  |  | *not included on panel* | | XRCC6 (KU70), XRCC5 (KU86), MRE11 |
| telomerase inhibitors |  |  |  |  | *not included on panel* | | TNKS, TNKS2, PINX1 |
| shelterin |  |  |  | TERF2IP (RAP1) | *not included on panel* | | TERF1, TERF2, TINF2, POT1, ACD (TPP1) |
| **Sustaining proliferative signalling** | | | | | | | |
| cellular proliferation |  |  |  |  |  | MKI67 (Ki-67) | PCNA |
| growth factors |  |  |  |  | *not included on panel* | | EGF, NGF, HGF, IGF1, IGF2 |
| transcription factors |  |  |  | MYCL, MXD1 | MYC |  | MYCN, MAX, MNT, MXI1, FOXM1 |
| DNA replication |  |  | TOP2B | GINS2, GINS3, GINS4, MCM2, MCM3, MCM5, MCM6, LIG1 | *not included on panel* | | GINS1, MCM4, MCM7, MCM8, MCM9, MCM10, CDC45, LIG3, POLA1, POLA2, POLB, POLD1, POLD2, POLD3, POLE, POLE2, POLE3, POLE4, POLG, POLH, POLI, POLK, POLL, POLM, POLN, POLQ, PRIM1, PRIM2, TOP1, TOP2A, TOP3A, TOP3B |
| serine/threonine-specific protein kinases (STKs) |  |  | BRAF, TGFBR3 | TGFBR1 |  |  | MTOR, ATM, CHEK2, IRAK1, TGFBR2, RAF1 (c-RAF), ARAF, AKT1 (PKB), AKT2, AKT3, BUB1 |
| RAS proteins |  |  |  | KRAS |  |  | HRAS, NRAS |
| mitogen-activated protein kinases |  |  | MAPK13, MAP3K11 | MAPK11, MAP3K9, MAP3K13, MAP4K1, MAP4K3, MAP4K5 |  |  | MAPK1 (ERK2), MAPK3 (ERK1), MAPK4, MAPK6, MAPK7, MAPK8, MAPK9, MAPK10, MAPK12, MAPK14, MAPK15, MAP2K1 (MEK1), MAP2K2 (MEK2), MAP2K3, MAP2K4, MAP2K5, MAP2K6, MAP2K7, MAP3K1, MAP3K2, MAP3K3, MAP3K4, MAP3K5, MAP3K6, MAP3K7, MAP3K8, MAP3K10, MAP3K12, MAP3K14, MAP3K15, MAP3K19, MAP3K20, MAP3K21, MAP4K2, MAP4K4, MAPKAPK2, MAPKAPK3 |
| receptor tyrosine kinases (RTKs) | IGF1R, EGFR (ERBB1) | ERBB3, ERBB4, DDR1, KIT |  | ROR2 | MET (HGFR), PDGFRA | ERBB2, RET | IGF2R, PDGFRB, ROR1, INSR, PTK7 (CCK7), AXL, SYK, ROS1, FLT3, NGFR |
| cyclins | CCND2, CCND3 | CCND1 |  |  |  |  | CCNA1, CCNA2, CCNB1, CCNB2, CCNE1, CCNE2 |
| cyclin-dependent kinases |  | CDK1 | CDK6 | CDK3 |  |  | CDK2, CDK4 |
| Hedgehog signalling |  |  | SMO |  | *not included on panel* | | DHH, SHH |
| Wnt signalling |  |  | FZD4, FZD8, WNT10A | DVL2 | *not included on panel* | | FZD1, FZD2, FZD3, FZD6, FZD7, FZD9, FZD10, WNT2, WNT2B, WNT3, WNT3A, WNT4, WNT5A, WNT5B, WNT6, WNT7A, WNT7B, WNT8A, WNT9A, WNT10B, WNT11, WNT16, DVL1 |
| **Evading growth suppressors** | | | | | | | |
| tumour suppressors | TP63 (p63), NDRG1 | NDRG2 |  |  |  | BRCA1 | TP53 (p53), RB1 (Rb), PTEN, VHL, APC, BRCA2, PTCH1, KLF6, RBL1, RBL2, NDRG3, NDRG4 |
| E2F transcription factors |  |  |  | E2F1, E2F7 | *not included on panel* | | E2F2, E2F3, E2F4, E2F5, E2F6, E2F8 |
| cyclin-dependent kinases inhibitors (CDKNs) |  |  |  |  | CDKN2B (p15^INK4B^) |  | CDKN2A (p16^INK4A^), CDKN2C (p18^INK4C^), CDKN2D (p19^INK4D^), CDKN1A (p21^CIP1^), CDKN1B (p27^KIP1^), CDKN1C (p57^KIP2^) |
| p53 inhibition |  |  |  | MDM1 |  |  | MDM2, MDM4 (MDMX) |
| **Deregulating cellular energetics** | | | | | | | |
| aerobic glycolysis (Warburg effect) |  |  | LDHA, SLC1A5 | IDH2 | SLC16A1 (MCT1) |  | SLC16A4, IDH1, LDHB, LDHC, GLS2, GLUD1 |
| hypoxia-inducible factors | HIF1A (HIF-1α) |  |  |  |  |  | EPAS1 (HIF-2α), NFE2L2 |
| **Resisting cell death** | | | | | | | |
| anti-apoptotic |  |  |  | BCL2L2 (Bcl-w) |  |  | BCL2, BCL2L1 (Bcl-XL), MCL1, BCL2A1, XIAP, BIRC2, BIRC3, BIRC5, BIRC7, CFLAR (FLIP) |
| pro-apoptotic | TNFSF10 (TRAIL) |  | CASP8, CASP9 | DAXX, BCL2L11 (BIM), BIK, BMF |  | FASLG, TNFRSF1B (TNFR2) | FAS, TNFRSF1A (TNFR1), TNFRSF6B, TRAF2, FADD, CRADD (RAIDD), CASP3, CASP6, CASP7, CASP10, APAF1, BAD, BID, BAX, BAK1, BBC3 (PUMA), PMAIP1 (NOXA), DIABLO (SMAC), HTRA2 |
| autophagy |  |  | PRKAA2 (AMPK) | PRKAG2, UVRAG |  |  | MAP1LC3B (LC3), ULK1, ULK2, BECN1 (Beclin-1), PRKAA1, PRKAB1, PRKAB2, PRKAG1, PRKAG3, RUBCN, ATG2A, ATG2B, ATG3, ATG5, ATG7, ATG9A, ATG9B, ATG10, ATG12, ATG13, ATG14, ATG16L1, ATG101 |
| pyroptosis |  |  |  | GSDMD |  |  | CASP4 |
| **Genome instability and mutation** | | | | | | | |
| Poly (ASP-ribose) polymerases (PARPs) |  |  | PARP4 | PARP1 |  |  | PARP2, PARP3, PARP6, PARP8, PARP9, PARP11, PARP12, PARP14, PARP15, PARP16 |
| DNA mismatch repair (MMR) proteins |  |  |  | MLH1 |  |  | MLH3, MSH2, MSH3, MSH6, PMS1, PMS2, MGMT |
| Genotoxic stress |  |  | GADD45A |  |  |  | GADD45B, GADD45G |
